# *In vitro* Evaluation of *Eclipta alba* against Serogroups of *Leptospira interrogans*

**DOI:** 10.4103/0250-474X.49124

**Published:** 2008

**Authors:** N. Prabhu, Joseph Pushpa Innocent, P. Chinnaswamy, K. Natarajaseenivasan, Lakshmi Sarayu

**Affiliations:** Postgraduate and Research Department of Microbiology, Dr. NGP Arts and Science College, Coimbatore-641 035, India; 1 Division of Microbiology, Rajah Muthiah Medical College and Hospital, Annamalai University, Chidambaram, India; 2 Institute of Laboratory Medicine, Kovai Medical Center and Hospital, Coimbatore-641 014, India; 3 Department of Microbiology, School of Life Sciences, Bharathidasan University, Tiruchirapalli-620 025, India

**Keywords:** Leptospirosis, *Leptospira interrogans*, *Eclipta alba* L., tube dilution, micro dilution

## Abstract

Leptospirosis is now acknowledged as the most widespread zoonoses in the world. Hundreds of cases occur in India every year accounting for considerable morbidity and sizable mortality. Several studies have delineated the epidemiology, pathology and variable clinical features of this condition. The present study comprises the importance and utilization of traditional based medicines to overcome the adverse reaction by conventional drugs and standardize the technology. The antileptospiral activity of *Eclipta alba* L. was well studied by both tube dilution and micro dilution techniques and the result showed better inhibitory action against various serogroups of *Leptospira interrogans. L. australis, L. autumnalis* and *L. grippotyphosa* are inhibited by both water and ethanol extract by tube dilution technique. The MIC level observed are 50 μg and 100 μg respectively. Similarly acetone extract, Icterohaemorrhagiae was responded to 200 μg/ml as MIC whereas in petroleum ether extract, no inhibition was observed. In the case of micro dilution technique, the entire inhibition rates are supported to the tube dilution technique. It showed that the micro dilution technique is the best method where we obtained the results within 30 minutes; at the same time tube dilution technique takes minimum of 7 days to provide the result.

Thousands of bacterial species have been isolated and studied, so far; the concept of natural focality has been developed in its relation to infections transmitted from animals to man and parasitic diseases. Infectivity or the ability to breach the new host's defenses and virulence, and variable that is multifactoral and denotes the capacity of a pathogen to harm the host^1^. India is a vast country having wide diversity in eco climatic conditions, botanical and mineral wealth, flora and fauna, it also have well sitting in a gold mine of well recorded and well practiced knowledge of traditional herbal medicines. Inspite of modern developments medical facilities, about 80% of Indian population are depend on traditional system of medicines because of severe adverse reactions by western medicines^2^. Herbal medicines are still the mainstay of about 75–80% of the world population, mainly in the developing countries, for primary health care. It has better cultural acceptability and compatibility with the human body and lesser side effects.

Leptospirosis is a contemporary, ubiquitous, zoonotic disease of worldwide in distribution which affects internal organs producing multiple organ dysfunction (MOD) to multiple organ failure (MOF), which is basically an occupational disease; man gets the infection by virtue of his occupation^3^. In 1888, this disease was reported among agricultural workers with some febrile illness. It also affects other occupational groups those who have a close proximity with the animals and water bodies^4^. Human infection is accidental, usually occurring after direct or indirect contact with urine from leptospiruric animals^5^. Other mechanisms like animal bites, handling of infected tissues, spreading via the ingestion of contaminated food and water are unusual. Leptospirosis is more common in southern parts of India and large numbers of outbreaks have been noticed during the period of October to December, every year in Tamilnadu.

An effective course of treating leptospirosis still remains conscious unsolved problem^6^. Leptospirosis usually responds to treatment with antibiotics, provided they are administered in enough doses early in the infection. Benzyl penicillin should be administered intravenously for upto 7 days in a daily dose of 6-8 mega units (3.6–4.8 g) but penicillin may cause a temporary exacerbation of the symptoms (Jarisch–Herxheimer reaction), but one should not prevent continuation of treatment^7^. Tetracycline should be administered if there is evidence of renal failure. Continuous renal replacement therapy is supposed to be superior to conventional hemodialysis in leptospirosis^8^. Vaccines are currently available in a very limited availability outside certain geographical areas and few are licensed in developed countries^9^. Leptospirosis affects internal organs producing MOD and MOF with a mortality rate of 10–20%. Every year during and after monsoon nearly 40% of leptospirosis cases are reported in Chennai and other parts of Tamilnadu. Mainly penicillin and doxycycline are the antibiotic of choice to cure the leptospirosis cases and the information about the role of medicinal plants against leptospirosis cases are scanty^10^. To overcome the adverse reaction by the above drugs, herbal-based therapeutics had been used in treating leptospirosis.

The efficacy of *Eclipta alba* against *L. interrogans* serogroups was investigated by evaluating the minimum inhibitory concentration (MIC) of the extracts like acetone, water and saponified lipid by using standard tube dilution in comparison with micro dilution technique^11^. In this study *Eclipta alba* L. was selected to identify the efficacy of antileptospiral capacity.

*Eclipta alba* grouped under the family *Asteraceae* that is of herbaceous type and spreads on ground or partly ascending with its stem and small leaves are succulent and are found mostly in tropical and subtropical regions where water logging condition is very high. Traditionally, this plant was used for curing the liver related problems. It is observed that the leptospirosis of main, severe and natal symptoms observed are jaundice (60%) and renal failure (20%) where jaundice play major role for increasing the mortality rate. In this study, an attempt was made to screen *Eclipta alba* to formulate antileptospiral drug.

The whole plant was collected from the agricultural fields and it was cleaned by soaking in the tap water to remove the soil and other debris. Then the leaves were separated and shadow dried. It was ground into tiny pieces and the bioactive compounds were extracted using various solvents like acetone, ethanol, petroleum ether and water. Extracted compound was diluted by using double distilled water. Various concentrations of compound were made ranging from 50, 100, 150, 200 and 250 μg in 1 ml and were used for antileptospiral activity.

Susceptibility testing was used to determine the minimal amount of plant extracts which would inhibit the maximum growth of leptospires MIC; kill *in vitro* (minimal leptospiricidal concentration- MLC) or (maximum lethal effect– MLE). The standard methods to be followed for the study of efficacy of drugs against leptospiral members are tube dilution technique (TDT) and micro dilution technique (MDT). The TDT was done by adding various concentrations of the compound in the Ellinghausen, McCullough, Jensen and Harris (EMJH) liquid medium. After sterility checking of the medium, a battery of 7 serogroups was inoculated with syringe filter. The tubes were incubated at room temperature for 7 days. The inhibition patterns for each serogroups in different concentrations were observed under dark field microscope.

In MDT, the viable leptospiral serogroups were taken in all the wells of micro titer plate (each row contain serogroups) and the diluted extracts were mixed in all the wells (each column contain separate concentration of the extracts). This culture extract mixture was well mixed and the microtitre plate was covered with clean aluminum foil and kept under dark condition for incubation at room temperature. After 30 min, the samples were spread on a slide by micro diluter and observed under dark field microscope to study the inhibition. In these techniques, the results were depicted the percentage of inhibition. The inhibition profile of various serogroups were tabulated and compared with control.

The results of various extracts of the *Eclipta alba* were described to improve the therapeutic values of the leptospirosis by various serogroups. The antimicrobial standardization of the spirochetal members was well studied in Tube dilution technique and the study was under diagnosed and screened in dark field microscope and the results was impregnated as percentages^12^.

In the case of TDT, on comparing with other solvents the water extract showed the better inhibitory property. The water extract concentration of 50 and 100 μg showed the better antileptospiral activity especially on the serogroups like Australis, Autumnalis and Grippotyphosa, remaining showed moderate reduction in numbers but Pomona having resistance capacity. The ethanol extract showed the 80% inhibition rate at 50 μg against Australis, Autumnalis, Grippotyphosa and Icterohaemorhagiae. No such reduction was observed in the remaining serogroups. The Autumnalis was inhibited by this plant extract at the concentration of 200 μg in the acetone extract. But no such observable result was countered by the petroleum ether extract.

In water extract under micro dilution technique, the minimum inhibitory concentration like 50 μg showed 100% inhibition against serogroups like Australis, Autumnalis and Grippotyphosa. Along with these serogroups, Icterohaemorrhagiae is also inhibited by the ethanol extract. All the inhibition rate of moderate level was observed same like tube dilution technique by ethanol extract. The highest concentration level of 250 μg having 100% inhibition rates against Autumnalis and Icterohaemorrhagiae. Same to the above technique, the petroleum ether extract having no such inhibition. The detailed inhibition rate by tube dilution and micro dilution technique was depicted in the Table 1.

**TABLE 1:** EFFECT OF VARIOUS EXTRACTS OF *ECLIPTA ALBA* AGAINST SELECTIVE SEROGROUPS OF *LEPTOSPIRA INTERROGANS*

Name of the serogroup (Leptospira interrogans)	Technique	Inhibition rate (%) in various concentration (μg) in different solvents																			
	
		Aqueous					Ethanol					Acetone					Petroleum ether				
				
		50	100	150	200	250	50	100	150	200	250	50	100	150	200	250	50	100	150	200	250
*L. australis*	MDT	100	100	100	100	100	100	100	100	100	100	-	-	-	-	-	-	-	-	-	-
	TDT	100	100	100	100	100	80	100	100	100	100	-	-	-	-	-	-	-	-	-	-
*L. autumnalis*	MDT	100	100	100	100	100	100	100	100	100	100	-	-	20	40	100	-	-	-	-	-
	TDT	100	100	100	100	100	80	100	100	100	100	-	20	40	100	100	-	20	20	20	20
*L. grippotyphosa*	MDT	100	100	100	100	100	100	100	100	100	100	-	-	-	-	20	-	-	-	-	-
	TDT	100	100	100	100	100	80	80	100	100	100	-	-	-	-	-	-	-	-	-	-
*L. canicola*	MDT	-	-	-	-	20	-	-	-	-	-	-	-	-	-	20	-	-	-	-	-
	TDT	20	20	60	80	80	-	-	-	-	20	-	-	-	-	20	-	-	-	-	-
*L. icterohaemorrhagiae*	MDT	-	-	20	40	60	80	100	100	100	100	-	20	40	40	100	-	-	-	-	-
	TDT	10	10	20	20	40	80	100	100	100	100	20	20	40	60	100	-	-	-	-	-
*L. hebdomadis*	MDT	-	-	-	-	-	-	-	-	-	20	-	-	-	-	-	-	-	-	-	-
	TDT	10	20	20	20	40	-	-	-	-	40	-	-	-	-	-	-	-	-	-	-
*L. pomona*	MDT	-	-	-	-	-	-	-	-	-	-	-	-	-	-	-	-	-	-	-	-
	TDT	-	-	-	-	20	-	-	-	-	20	-	-	-	-	-	-	-	-	20	40

Inhibition rate of MDT and TDT of various extracts of *Eclipta alba* against selective serogroups of *Leptospira interrogans*. MDT is micro dilution technique and TDT tube dilution technique

The bioactive principle from *Eclipta alba* was extracted by various solvents and it was get ready for inoculation on the tubes with already standardized leptospiral cultures. By following the periodic observation^13^ of the tubes under dark field microscopy, the inhibitory activity of the plant compound was easily detected by the reduction in the numbers and its motility of the leptospires compared with control (without plant extract). The lowest concentration level of 50 μg itself showed the complete reduction in water extract against certain serogroups of leptospires and this much inhibition level is not observed in any solvents used under this study. On comparing with the efficacy of *Phyllanthus niruri*^11^, the *Eclipta alba* showed the better result by water extract. The ethanolic extract showed the accurate inhibitory results same like aqueous extract. So, the MLC by both the techniques of ethanol extract was 100 μg.

There is a saying evidence of the usage of acetone as a sole solvent for extracting the bioactive compounds of plant origin and study the antileptospiral study, which provide renewable results of reduction in numbers^1^. To think over in this mind, the extraction of the bioactive compounds of *Eclipta alba* by using acetone was studied against *L. interrogans* serogroups. The acetone extracted *Phyllanthus niruri* was already studied and proved as best antileptospiral agent but *Eclipta alba* showed moderate reduction when compared with aqueous and ethanol basis.

On comparing with tube dilution technique, micro dilution was found to be better that it cleared the leptospires even during the study period of 30 min, which might make this method to be better suited for performing antileptospiral studies^14^. By observing all the above results, the *in vitro* antileptospiral activity of *Eclipta alba* was well studied and proved as a best antileptospiral drug. As per this investigation, it is confirmed that the bioactive compounds of *Eclipta alba* L. may be used as an antimicrobial; agent for leptospiral infection both for prophylaxis and treatment, but it should be further confirmed by *in vivo* experiments and field trials.

On comparing with other serogroups, the members like Australis, Autumnalis, Grippotyphosa and Icterohaemorrhagiae are inhibited by various extracts of *Eclipta alba.* This is first kind in antimicrobial history of introducing and analyzing *Eclipta alba* against Leptospirosis. It provides an idea in the improvement of medicinal herbs against leptospiral members to overcome the adverse reaction like Jarisch-Herxheimer reaction and also identify the presence of bioactive compounds in the *Eclipta alba* as therapeutics. It should be further investigated for the type and nature of compound in it which inhibit the *Leptospira interrogans* serogroups.
